# A novel method for extracting metals from asteroids using non-aqueous deep eutectic solvents

**DOI:** 10.1038/s41598-023-44152-0

**Published:** 2023-10-08

**Authors:** Rodolfo Marin Rivera, Philip Bird, Gawen R. T. Jenkin, Andrew P. Abbott

**Affiliations:** 1 Centre for Sustainable Material Processing, School of Chemistry, University of Leicester, Leicester, LE1 7RH UK; 2 Centre for Sustainable Resource Extraction, School of Geography, Geology and the Environment, University of Leicester, Leicester, LE1 7RH UK

**Keywords:** Geochemistry, Mineralogy, Chemical engineering, Catalysis, Chemical engineering, Materials chemistry, Green chemistry, Ionic liquids

## Abstract

Extra-terrestrial mining and metal processing are vital for access to strategic metals for space exploration. This study demonstrates for the first time the catalytic dissolution of metals from meteorite proxies of metal-rich asteroids using a deep eutectic solvent (DES). DESs are of particular interest for extra-terrestrial mining as they can be designed to have relatively low vapour pressures and could potentially be made from organic waste products created in extra-terrestrial settlements. Three types of meteorites were investigated: two chondrites (H3, H5) and one iron (IAB-MG) meteorite. Chondrite samples were composed of silicates (olivine, pyroxene) with metal-rich phases occurring as native metal alloys, sulphides and oxides. Metallic Fe–Ni and troilite (FeS) are the most abundant metal-bearing phases in all three samples, particularly in the iron-rich meteorite. The samples were subjected to chemical micro-etching experiments with iodine and iron(III) chloride as oxidising agents in a DES formed from the mixture of choline chloride and ethylene glycol. Micro-etching experiments demonstrated that Fe–Ni rich phases are effectively leached out in this system, while other mineral phases remain unreactive.

## Introduction

The establishment of viable extra-terrestrial settlements will require local sources of raw materials for construction and in situ technology production and, key to these, is the ability to extract metals. The efficient utilisation of local materials and resource recovery from space could significantly reduce the mass, cost and environmental constraints imposed on space missions^1–3^. The lunar regolith has large amounts of oxygen, silicon and metals^4,5^, while near-Earth asteroids and comets can contain substantial amounts of metals, oxygen, hydrogen, carbon and, in some cases, also nitrogen. In fact, some of the largest known metal-rich asteroids are located in the middle and outer part of the asteroid belt, between the orbits of Jupiter and Mars, at 2.7–3.0 au from the sun (4.0–4.5 × 10^8^ km). These large metal-rich asteroids have been considered as the parental bodies of iron meteorites, enstatite chondrites, stony-iron, and metal rich carbonaceous chondrites^6–8^. Near Earth asteroids (NEAs) can contain valuable platinum group metals (PGMs), as well as iron, nickel and cobalt in concentrations greater than on the Earth’s surface^9,10^. For instance, the spectral signature of asteroids 1986 DA and 2016 ED85 are quite similar to asteroid 16 Psyche^11^, the largest metal-rich body in the solar system (radius of ca. 113 km). Both NEAs have surfaces with 85% metal (mostly iron, cobalt and nickel) and 15% of silicate material^12^. These metals, if recovered, could provide a ‘local’ source of materials essential for establishing a human settlement in space or on other terrestrial bodies. PGMs, for example, can be used as catalysts to enhance oxygen and hydrogen production, whereas iron, cobalt and nickel can be used as raw materials for infrastructure. Asteroids can also have a significant concentration of selenium and tellurium^13,14^, which can be used as raw materials for the construction of solar panels, fuel cells, thermoelectric generators, energy storage and/or organic synthesis^15–18^.

Over the last 60 years, different process technologies have been conceptualised and demonstrated for oxygen and hydrogen production from lunar regolith and Martian soil^1,4,19–25^. Although the extraction of rare earth elements from basalt rocks using microorganisms in different gravity regimes has recently been demonstrated on the International Space Station^26^, the recovery of metals has generally been treated solely as a by-product rather than a target resource. The Fray-Farthing-Chen (FFC) Cambridge process considers the solid-state electrochemical reduction of metal oxides to metals in molten salt (e.g., CaCl_2_) at temperatures above 900 °C^27–29^. This method can enable both the extraction of oxygen (up to 45%) from lunar regolith and the simultaneous production of metal alloys, such as Al/Fe, Fe/Si, Ca/Si/Al, as by-products^30^. Similarly, carbothermic reduction at 1,120 °C with carbon formed by cooling carbon monoxide produced from carbon dioxide electrolysis can enable the extraction of iron alloys from Martian soil^31^. Although, the proposed methodologies have shown great potential for oxygen production as the main goal, they also have significant drawbacks. Mineral processing operations are needed firstly to concentrate the mineral phases of interest and reject the unwanted material. The energy consumption of these processes is expected to be around 50 GJ/tonne of metal, which accounts about 45% of the total energy spent on modern mining on Earth^32^. It must be noticed that contrary to the vast utilisation of fossil fuels as an energy source for mining on Earth, solar energy will be the primary power source in space^33^. Furthermore, whilst the electrochemical reduction process of solid compounds in high temperature molten salts has only been demonstrated at a laboratory scale^34^, it is clear that the technology will need the continued re-supply of suitable electrodes, most likely from Earth, as they are normally made of carbon or graphite which have limited potential for in-space production. In addition, similarly to traditional pyrometallurgical processes, e.g., calcination, roasting and smelting, a robust infrastructure in terms of shell and lining structure will also be necessary for working in high temperature operating conditions. The high-temperatures proposed in these technologies also constitute a significant safety concern and require extensive, expensive encapsulation and thermal management systems to prevent overheating in low gravity environments with little or no convective dissipation. Consequently, the development of cost-efficient, low-temperature, simple and safe extraction/recovery processes that can be implemented in space is still desirable. Until now, comminution, beneficiation and metal production have all been developed and refined over time with base assumptions that come inherently from the physical and chemical conditions found on Earth, and they cannot be directly implemented in space^35^.

In order to meet the energy challenges for in situ resource utilisation (ISRU) deployment^2^, electrochemical methodologies driven by solar photovoltaic energy conversion represents the most promising methodology for minerals and metals processing. Electrochemical methods have already been demonstrated as an alternative methodology for solubilising metal oxides in non-aqueous ionic solvents such as deep eutectic solvents (DESs)^36^. DESs have been widely studied in different applications, but mostly in metal processing and electrodeposition^37^. They have the great advantage of being simple to prepare by combining a Lewis base and acid. They also have low toxicity, are not flammable, and can have low vapour pressures^38,39^ capable of enduring the hard vacuum of space while remaining in the liquid phase. Being water-free they reduce dependence on what will inevitably be a limited resource. Reactions are generally accomplished at low temperatures (20–80 °C), significantly reducing encapsulation and thermal challenges compared to high temperature processes. The most commonly investigated systems have been quaternary ammonium salts with hydrogen bond donors (HBD) such as urea, ethylene glycol and glycerol^40^. The utilisation of inorganic chloride salts-based DESs have also been investigated^41–44^, which opens the opportunity to synthesise novel DESs by utilising *in space* materials. The presence of calcium, aluminium, iron, and zinc, for instance, have already been reported in regolith^4,25,45^, whereas the presence of halogen gas (chlorine, bromine) have been reported in the atmosphere of some planets like Mars and Venus^46^. Urea and glycerol could also be produced in space from organic waste and therefore, be considered as potential HBDs^47–51^.

This work aims to investigate the proof-of-concept of a methodology for extracting metals from meteorite proxies of asteroids using non-aqueous deep eutectic solvents (DESs). Two types of chondrite meteorite (NWA 13876 and NWA 7160) and one iron-rich meteorite (Campo del Cielo) were investigated. The occurrence of minerals and metal phases was investigated by automated SEM–EDX analysis. The samples were subjected to chemical micro-etching experiments with iodine and iron(III) chloride as oxidising agents in a DES formed from the mixture of choline chloride (ChCl) and ethylene glycol (EG). Etching depth and dissolution rates were determined by analysing the 3D topography of the samples before and after etching.

## Results and discussion

### Meteorite characterisation

Overall, chondrite meteorites are characterised by their diverse mineralogy. Olivine, pyroxene, plagioclase and kamacite tend to be the most prominent minerals, with composition depending on the degree of metamorphism to which they were subjected^52,53^. In this research, the mineral constituents of the meteoritic samples were determined by automated SEM–EDX mapping and correlated to high resolution reflected light images. Bulk analysis of meteorites can be found in Tables S1, S2 and S3 as Supplementary Material.

**Figure 1:**
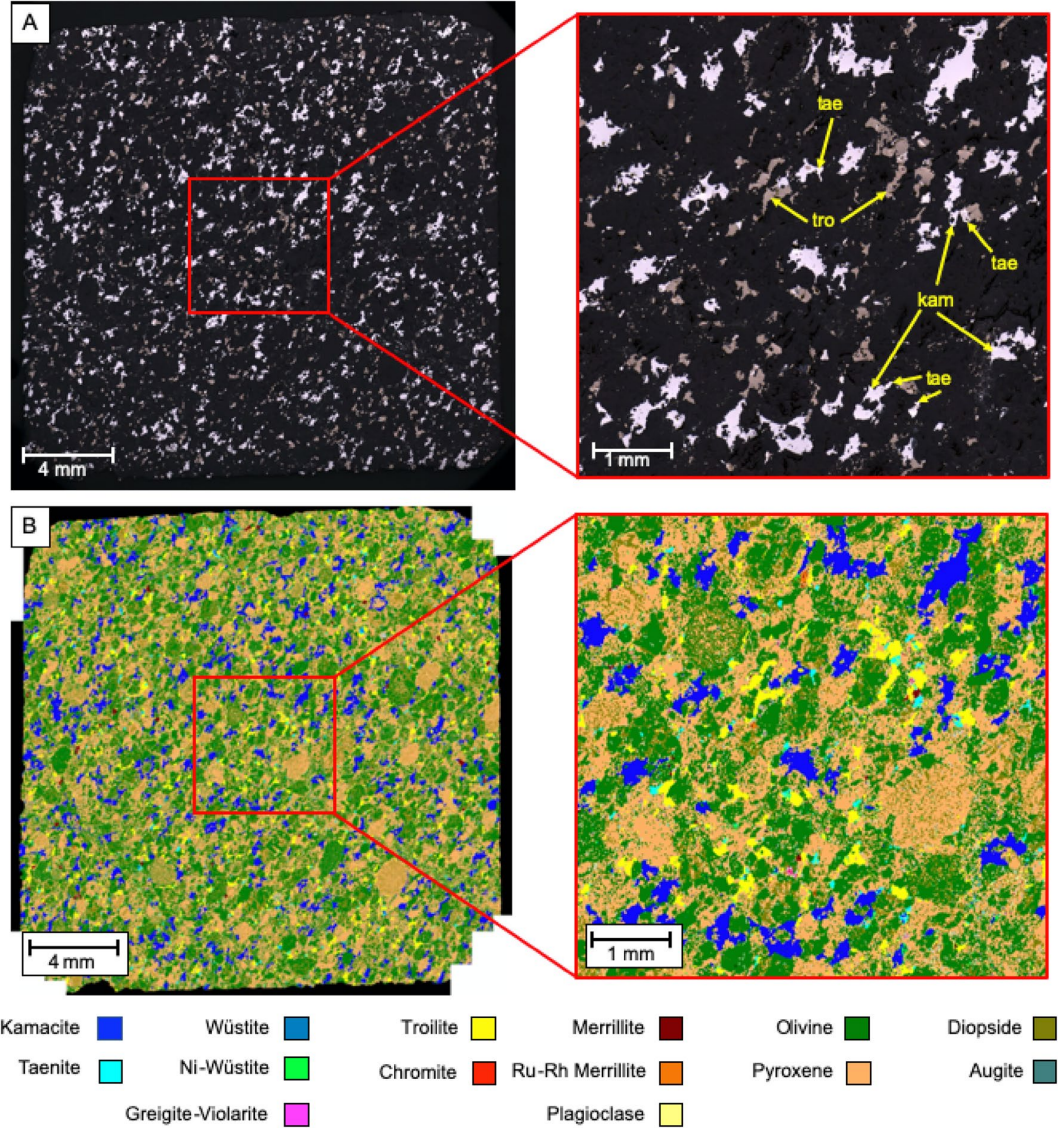
(**A**) Reflected light images of meteorite NWA 13876 (tae: taenite, tro: troilite, kam: kamacite) and (**B**) corresponding false colour mineral map generated by SEM–EDX analysis.

Meteorite NWA 13876, a H5 type chondrite, contains abundant Fe and Ni-rich phases with iron-nickel alloys (high reflectance white–grey), iron sulphide (moderate reflectance yellows) and minor iron-oxides (low reflectance grey) being observable (Fig. 1). These metal-rich phases occur as highly irregular, slightly elongated, space filling masses distributed throughout the silicate groundmass (dark grey), and are observed wrapping around the deformed chondrules within the sample. The silicate matrix is composed of chondrule forming pyroxene ((Mg, Fe, Ca)(Mg, Fe, Al)(Si,Al)_2_O_6_, 36 wt.%) and olivine ((Mg,Fe)_2_SiO_4_, 34 wt. %) with minor plagioclase feldspar (NaAlSi_3_O_8_ – CaAl_2_Si_2_O_8_, < 1 wt.%). Fe and Ni-rich phases account for 22 wt.% of the NWA 13876 sample; major phases are Fe–Ni alloys kamacite (94 wt.% Fe, 6 wt.% Ni) constituting 14 wt.% of the sample, and taenite (63 wt.% Fe, 37 wt.% Ni) constituting 1.5 wt.% of the sample, troilite (Fe_(1-x)_S with x = 0 to 0.2) constituting 5 wt.% of the sample, and nickeloan-wüstite ([Ni]-FeO) constituting 1 wt.% of the sample. Other trace metal-rich phases totalling less than 1 wt.% include wüstite (FeO), greigite-violarite (Fe^2+^Fe_2_^3+^S_4_–Fe^2+^Ni_2_^3+^S_4_) and chromite (FeCr_2_O_4_). The Ca-phosphate merrillite (Ca_9_NaMg(PO_4_)_7_) accounts for less than 0.5 wt.% of the sample, but is notable for a sub-variety that contains significant Ru (~ 6 wt.%) and Rh (~ 3 wt.%). Similar major mineralogical phases, i.e. olivine, pyroxene and plagioclase, were identified in a H5/6 ordinary chondrite found in Denmark, but their concentration (19, 21 and 10 wt.%, respectively) were much lower than the one hereby reported^54^.

**Figure 2:**
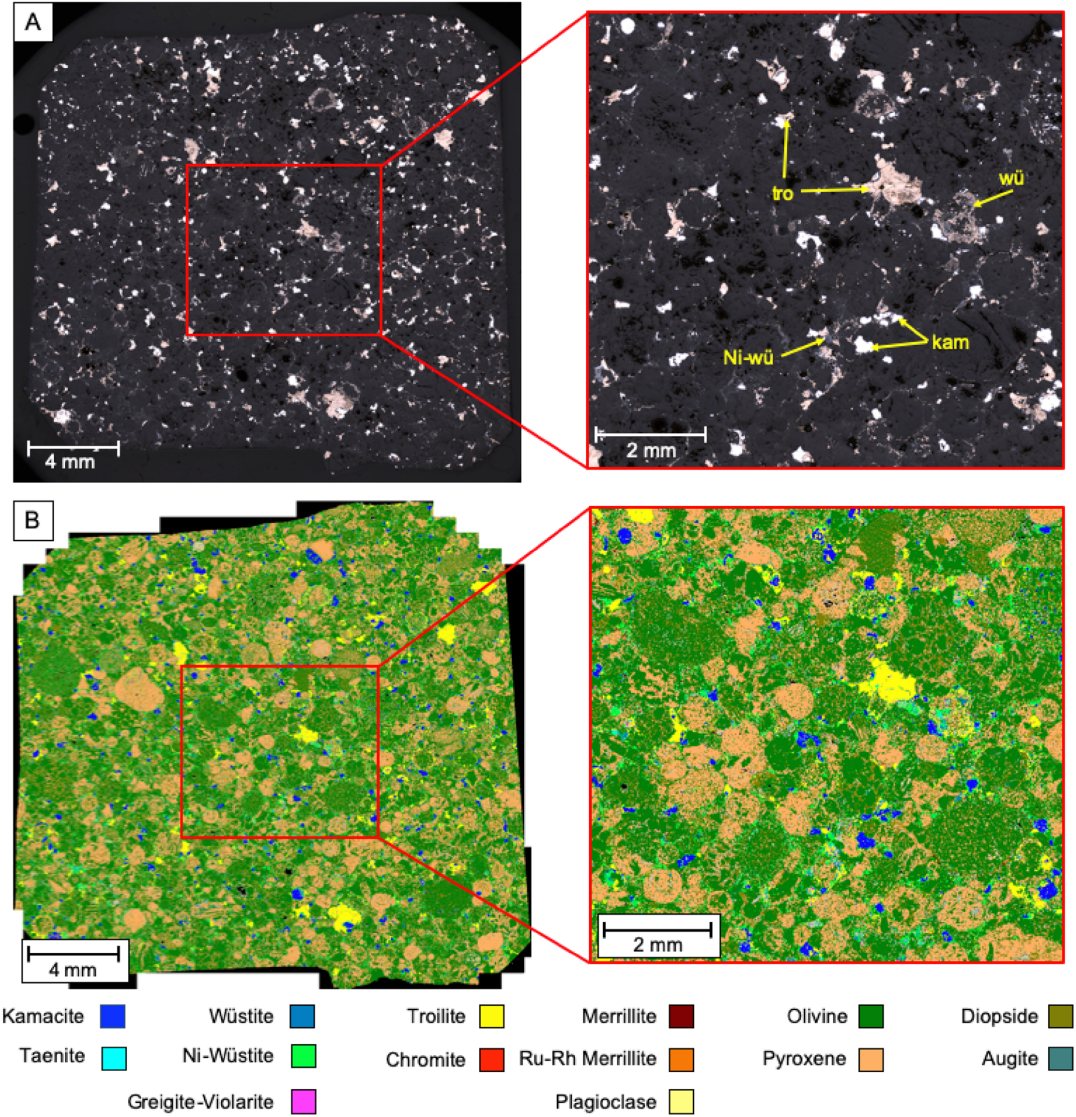
(**A**) Reflected light images of meteorite NWA 7160 (tro: troilite, kam: kamacite, wü: wustite, Ni-wü: nickeloan-wüstite) and (**B**) corresponding false colour mineral map generated by SEM–EDX analysis.

Sample NWA 7160, a H3 type chondrite, again contains iron-nickel alloys, iron sulphide and iron oxide minerals though is overall less abundant in Fe–Ni-rich minerals (16.7 wt.%), and contains a higher proportion of iron sulphide (Fig. 2). These metal-bearing phases form halos around the chondrules and in places within the chondrules forming sub-millimetre exsolution lamellae. The silicate matrix is composed of chondrule forming olivine (46 wt.%) and pyroxene (27 wt.%) with trace plagioclase feldspar. Metal-rich phases consist of nickeloan wüstite (7 wt.%), troilite (5 wt.%) and kamacite (88 wt.% Fe, 12 wt.% Ni) forming 4 wt.% of the sample with trace metallic phases totalling < 1 wt.% including wüstite, taenite (73 wt.% Fe, 27 wt.% Ni) and chromite. Calcium phosphate (merrillite) was again identified with the Ru-Rh sub-variety also being present. According to the literature, chondrules of H3 chondrites tends to be classified petrographically into six textural types: barred olivine, porphyritic olivine, porphyritic pyroxene, barred pyroxene, radiating pyroxene and fine-grained. Their composition, however, tends to be widely disperse in the different chondrules^55^.

**Figure 3:**
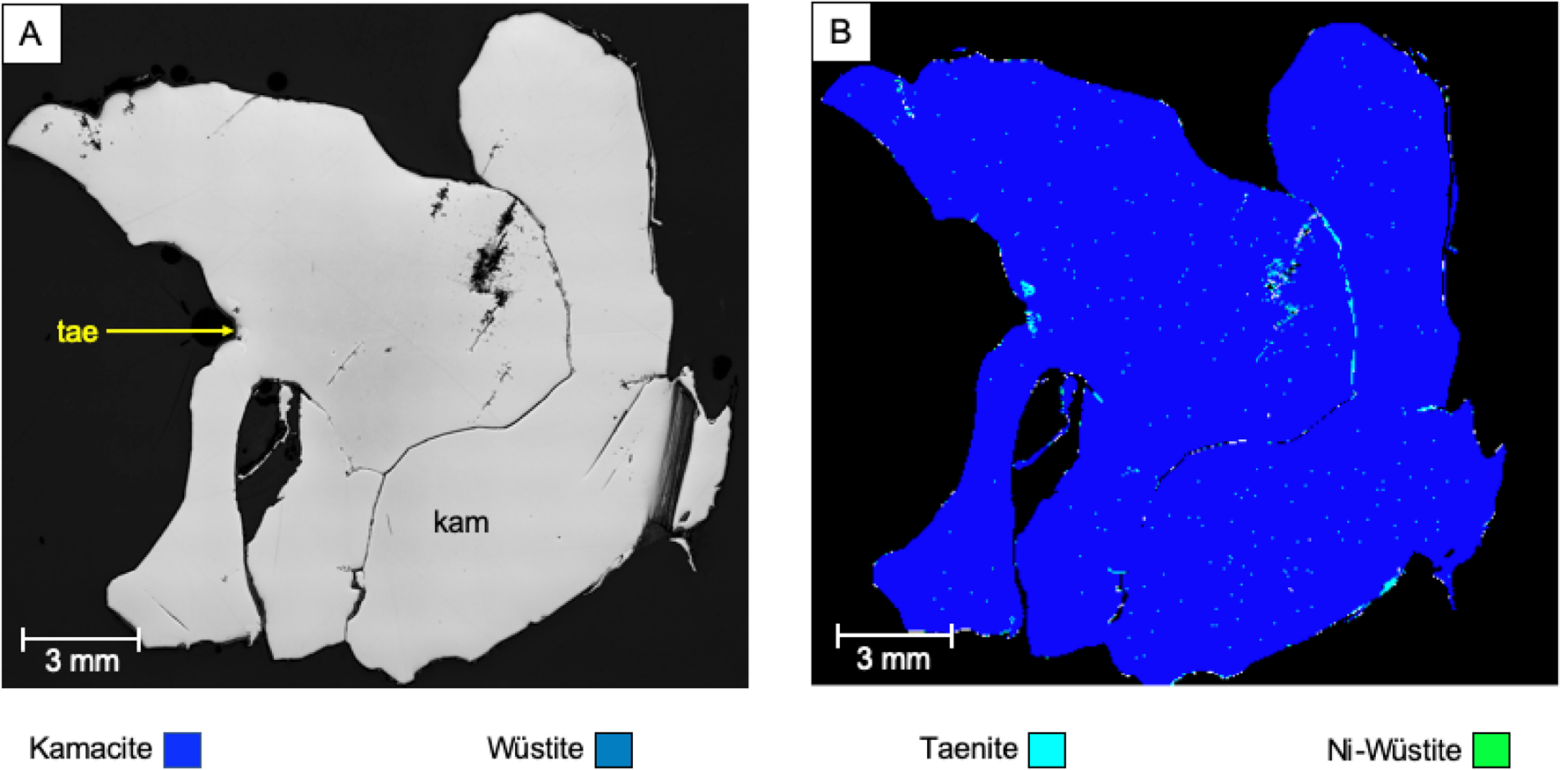
(**A**) Reflected light images of meteorite ‘Campo del Cielo’ (tae: taenite, kam: kamacite) and (**B**) corresponding false colour mineral map generated by SEM–EDX analysis.

The Campo del Cielo sample consists predominantly (> 99%) of kamacite (95 wt.% Fe, 5 wt.% Ni) (Fig. 3) with traces (< 1%) of taenite (66 wt.% Fe, 34 wt.% Ni), wüstite (FeO) and nickeloan-wüstite ([Ni]-FeO). Haubner and Strobl (2021) have classified this meteorite as a kamacite fragment containing 5.3 wt.% of Ni together with some traces of graphite and non-metallic phases^56^. Kamacite has also been documented to contain significant trace element components, such as Re, Os, Ir, Pt and Au^57^.

**Figure 4:**
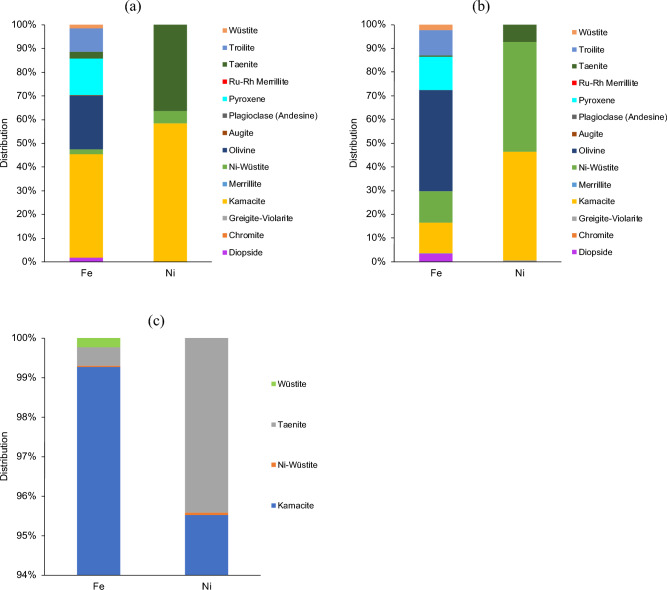
Mineral distribution of iron and nickel as determined by automated SEM–EDX analysis in (**a**) NWA 13876, (**b**) NWA 7160 and (**c**) Campo del Cielo.

Figure 4 shows the mineral distribution of iron and nickel in the studied meteoritic samples. In both the chondrite samples, iron is distributed across a range of silicate and Fe–Ni rich mineral phases. In sample NWA 13876 the bulk of the contained iron is hosted in Fe–Ni rich phases such as kamacite (43%), troilite (10%), taenite (3%) and Ni-wüstite (2%) though a significant amount also occurs in silicate phases including olivine (23%) and pyroxene (16%). Nickel is hosted exclusively in the Fe–Ni rich minerals with kamacite (58%), taenite (36%) and Ni-wüstite (5%) being the major mineral hosts. Sample NWA 7160 contains a higher proportion of silicate minerals, and this is reflected in the distribution of target metals. Iron is hosted mainly in silicate phases including olivine (43%), pyroxene (14%) and augite (3%), while Fe–Ni minerals contain a lower proportion with kamacite (13%), Ni-wüstite (13%) and troilite (11%) being most notable. The Campo del Cielo sample, as an iron-nickel meteorite, is composed entirely of Fe–Ni rich mineral phases, with kamacite hosting the majority of both iron (99%) and nickel (95%), with the remaining nickel being hosted in taenite (4%). The elemental distribution in the different mineral phases can be found in Fig. S2 in the Supplementary Material.

### Redox catalysts in DESs—a brief summary

The catalytic oxidation of metals has already been demonstrated for several solids, such as superalloys and minerals^58,59^, as well as in the recycling of spent electronic devices^41,60^. The advantage of electrocatalysis is that no direct contact is required between the anode and the material to be oxidised, and the chemical oxidising agent can be re-generated in situ by an electrochemical reaction (if it has a reversible electrochemical behaviour)^61^. While electrocatalysis can be carried out in a variety of media, non-aqueous eutectic mixtures can decrease passivation of some metals, while careful selection of the redox catalyst can enable selective solubilisation of metals from complex mixtures^37,61^. For instance, iodine has been reported as a strong oxidising agent for metal and mineral digestion, particularly for solubilising gold in a wide variety of solvents, both aqueous and non-aqueous systems^58,59,61–68^. Iron(III) chloride (FeCl_3_) and copper(II) chloride (CuCl_2_) have also been reported as redox catalysts in different ionic fluids and brines, for the dissolution of metals from solar photovoltaic cells and printed circuit boards. Both of them tend to have high solubility and fast electron transfer^41,60^.

The ChCl: 2EG system was considered as a solvent for processing the meteorites as has a relatively low viscosity. However, there would be other similar, non-aqueous, ionic fluids which would be more suitable for extra-terrestrial applications^69^. A general overview of the physical properties of this particular eutectic solvent, as well as its constituents, is shown in Table 1.

**Table 1:** General physical properties of the ChCl: 2EG eutectic solvent and its constituents (data has been adapted from^38,39,70–72^).

Property	ChCl	EG	ChCl: 2EG
Phase*	Solid	Liquid	Liquid
Molar mass, g mol^–1^	139.6	62.1	263.8
Melting point, °C	302	−12.9	< 40
Viscosity, mPa s	n.d.a	18.4 (20 °C)	20 (40 °C)
Density, g cm^–3^	1.02	1.11	1.12
Vapour pressure, mmHg	n.d.a	0.06 (20 °C)	29.9 (30 °C)
Electrical Conductivity, mS cm^–1^	15**	1.43 (30 °C)	7.12 (25 °C)

*n.d.a.* no data available.

*At 20 °C and 1 atm.

**ChCl dissolved in 20 wt.% H_2_O at 20 °C.

Chemical etching was investigated for all three samples using iodine and FeCl_3_, separately, as redox catalyst at a concentration of 0.1 mol dm^-3^. Iodine was selected due to its strong oxidative capacity, whereas FeCl_3_ was chosen due to the high concentration of iron in the samples. Notice that both redox catalysts have high solubility and display a reversible redox behaviour^61,73^, which can be explained by the following redox processes:

1 $$Fe^{III} + e^{ - } \leftrightarrow Fe^{II}$$

2 $$3I_{2} + 2e^{ - } \to 2\left[ {I_{3} } \right]^{ - }$$

3 $$\left[ {I_{3} } \right]^{ - } { } + 2e^{ - } \to 3I^{ - }$$

4 $$\left[ {I_{2} Cl} \right]^{ - } + 2e^{ - } \to 2I^{ - } + Cl^{ - }$$

### Chemical etching of chondrite meteorites

**Figure 5:**
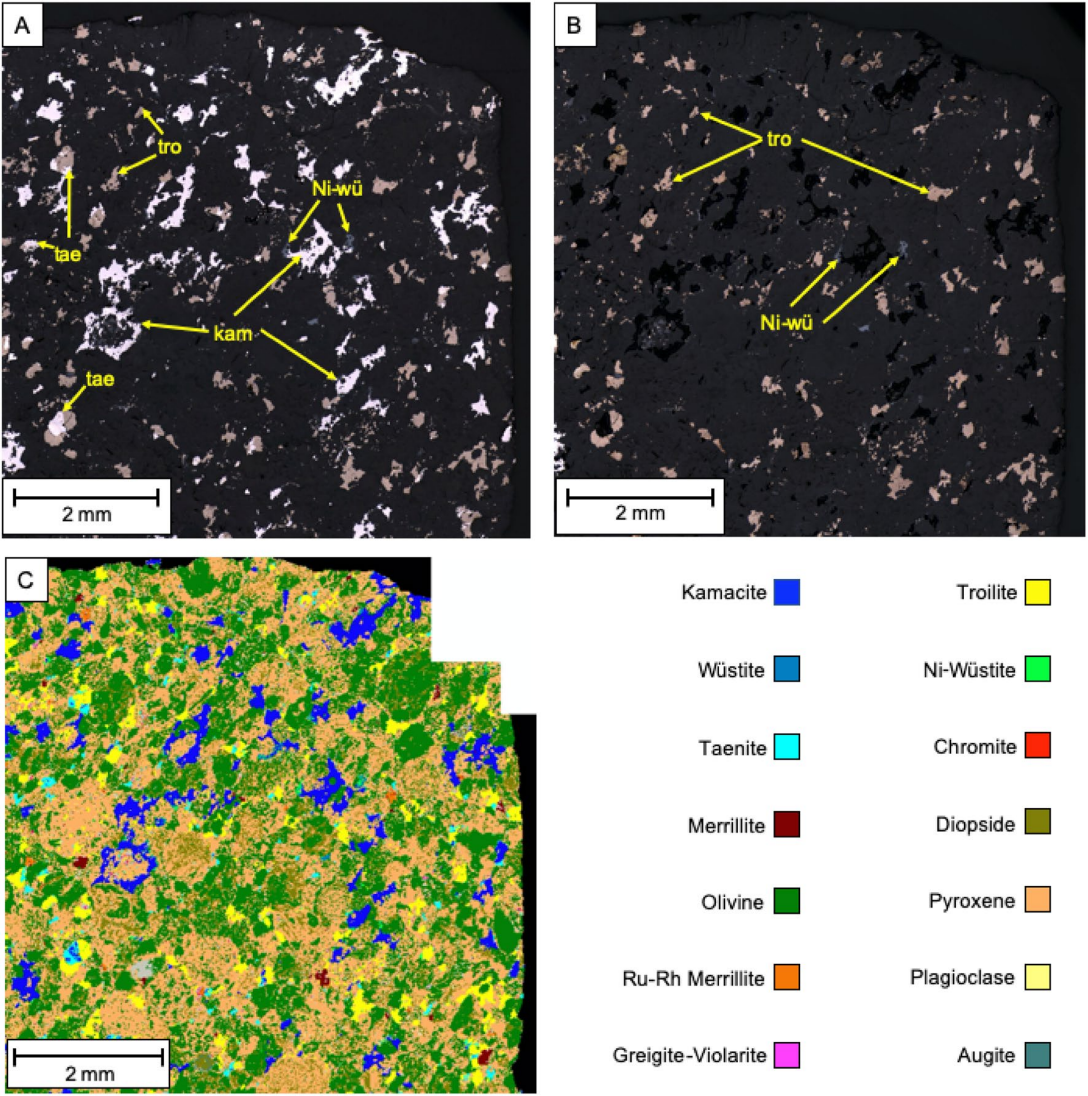
Reflected light images of meteorite NWA 13876. (**A**) Sample before chemical etching and, (**B**) same sample after 60 min of chemical etching with 0.1 mol dm^−3^ iodine as oxidising agent in ChCl: 2EG eutectic solvent. The corresponding distribution of minerals as determined by SEM–EDX is shown in (**C**).

The reflected light images of meteorites NWA 13876 and NWA 7160 before and after chemical etching in the presence of iodine and FeCl_3_ can be seen as Supplementary in Figures S3 and S4, respectively. A large portion of the surface exposed to the catalytic solutions, consisting of silicate materials (e.g., olivine), did not show signs of chemical oxidation in either sample. Figure 5 shows that chemical etching was observed for the kamacite and wüstite, with weak etching also in the taenite. The troilite, however, developed a yellow tarnish colour possibly due to the generation of sulphur or a passivating species able to change the optical properties of the underlying mineral. Such behaviour has already been observed when processing sulphide minerals with the same eutectic solvent^74–76^. The effect of iodine and FeCl_3_ as redox catalysts seems to have a similar effect, as both of them preferentially etched the Fe–Ni/Fe–O mineral phases over other mineral compounds.

**Figure 6:**
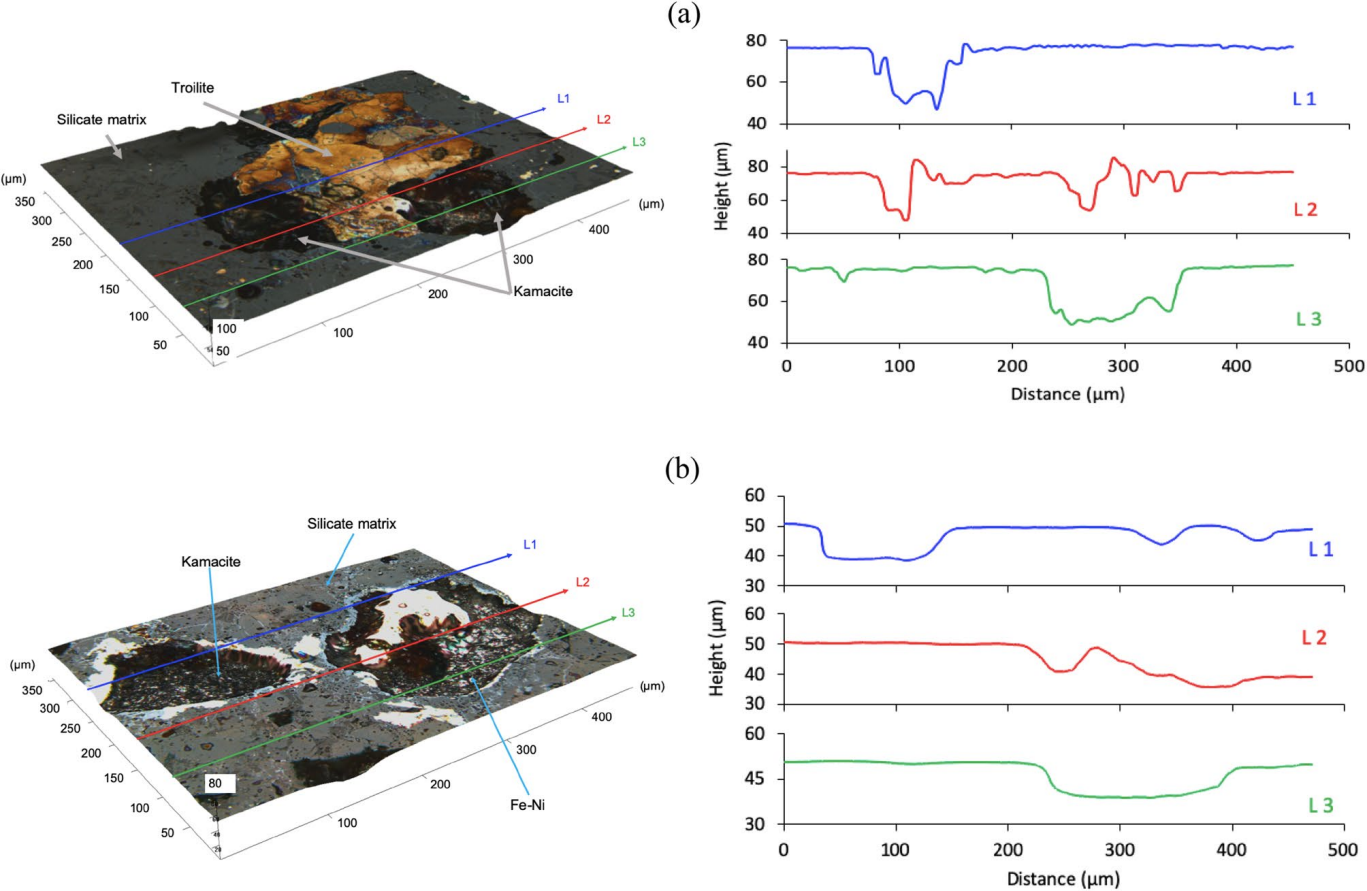
Etch depth in sample references (**a**) NWA 13876 and (**b**) NWA 7160, after 60 min of etching with 0.1 mol dm^−3^ iodine as oxidising agent. In the left-hand side, the 3D reflected light images (with line profiles) are described, whereas the topographic line profiles are shown in the right-hand side of the corresponding 3D image.

Figure 6 shows the etching depth in meteorites NWA 13876 and NWA 7160 after chemical contact with iodine in the ChCl: 2EG eutectic solvent. Both 3D images confirmed that Fe-Ni minerals were preferentially etched. The average depth obtained in these particles, after 1 h of experiment, was 33 ± 3.3 and 13 ± 1.3 μm for NWA 13876 and NWA 7160, respectively. These minerals were etched at an average rate of ca. 0.4 μm min^–1^, which was comparable to the rate obtained with FeCl_3_. The difference in etching rates was due to the distribution of iron within the difference mineral phases. In sample NWA 7160, iron was mostly concentrated in unreactive silicate minerals, such as olivine, pyroxene and augite, whereas in sample NWA 13876 iron was mostly concentrated in more soluble Fe–Ni rich phases, such as kamacite, troilite, taenite and Ni-wüstite (Fig. 4). The dissolution of nickel could also have contributed to the difference in etching rates, as it is known that it can form complexes either with the anion or with an oxygen donor (usually in the form of OH^-^ ions) of the DESs depending on its concentration and temperature^44^. For instance, nickel can form complexes with EG molecules, i.e. [Ni(EG)_3_]^2+^, whereas trichloro complexes with two or three hydrate water molecules can also be formed in the presence of water molecules (water content > 5 wt.%)^77,78^. Such complexes are likely to develop passivating layers on the surface of the particles^79^.

### Chemical etching of “Campo del Cielo” meteorite

**Figure 7:**
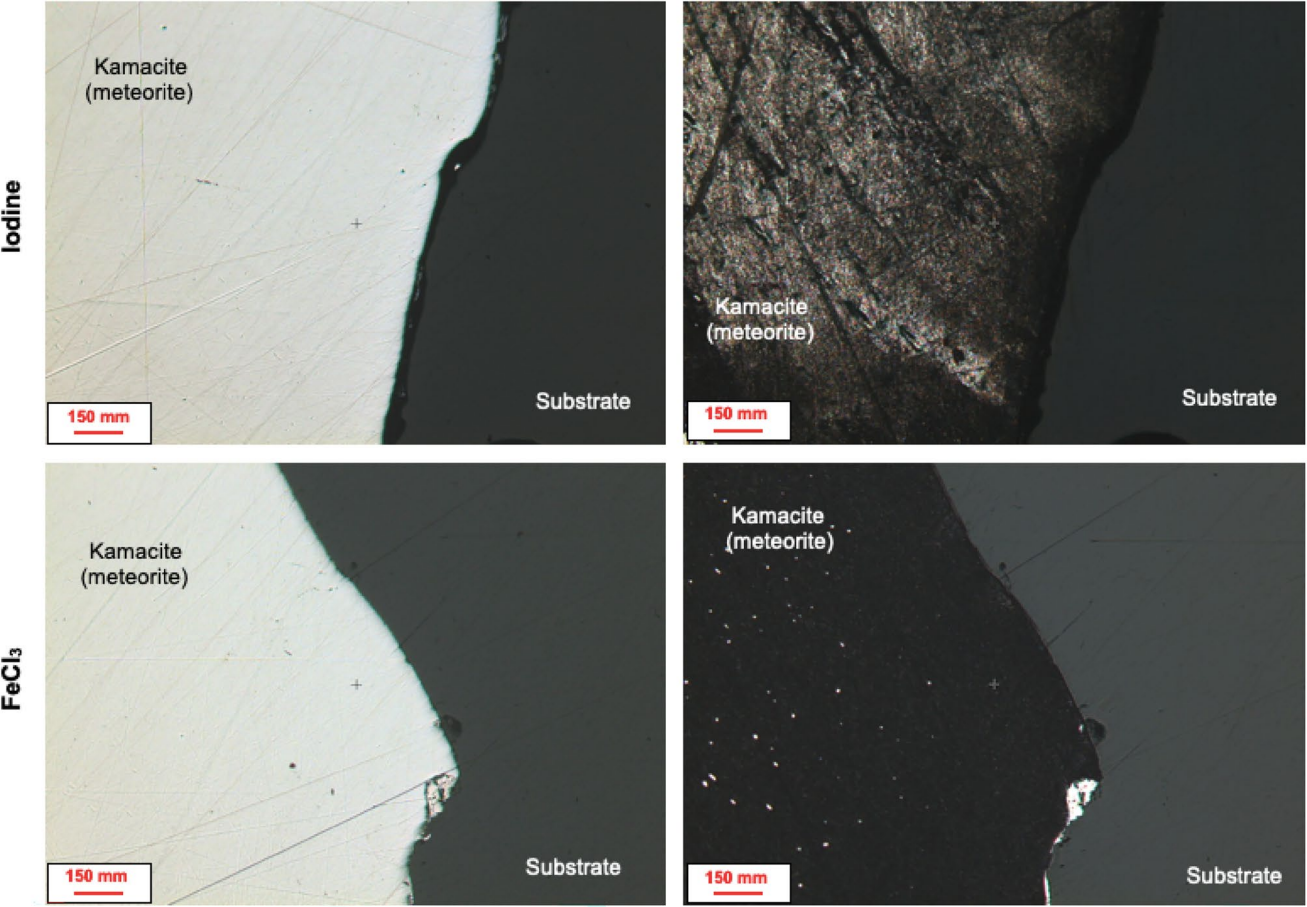
Reflected light images of the Campo del Cielo meteorite before and after 60 min of chemical etching with 0.1 mol dm^−3^ of iodine or FeCl_3_ as oxidising agents in ChCl: 2EG eutectic solvent.

Figure 7 shows the reflected light images of the Campo del Cielo meteorite before and after chemical etching with iodine and FeCl_3_ as oxidising agents. A dissimilar etching effect was observed on the sample. It is known that the behaviour of iron species in this DES is particularly interesting since only the Fe^III/II^ couple is stable^58^. As kamacite, a mineral composed principally of iron, is the major constituent in this sample, in the presence of FeCl_3_, the contained iron can be dissolved into the solution as Fe^II^ species (Eq. 5). In theory, part of the Fe^II^ species will be re-oxidised over time into Fe^III^ as long as there is sufficient oxygen present in the surrounding medium (Eq. 6). However, the development of both chemical reactions will be restricted by the presence of Fe^III^ in the solution. Eventually, Fe^II^ could have been subjected to secondary chemical reactions by forming a stable oxide/hydroxide complex, leading to the formation of a passivating film^79,80^. This could explain the dullness of the sample after etching. The formation of such a complex could hinder both the re-generation of Fe^III^ and the dissolution of metallic iron.

5 $$Fe^{III} + Fe^{0} \to 2Fe^{II}$$

6 $$Fe^{II} + O_{2} \to Fe^{III}$$

In the case of iodine, it has been reported that both [I_2_Cl]^−^ and [I_3_]^−^ are stable in ChCl: 2EG eutectic mixture (distribution between complexes tending to change as function of water addition)^81,82^, suggesting that both species should be able to oxidise iron according to Eqs. (7, 8). However, both the Fe^III/II^ and iodine redox couples are at very similar potentials in ChCl: 2EG^58^, suggesting that Fe^II^ can also be oxidised to Fe^III^ by iodine. This can explain the differences between the surface morphologies obtained after etching, as in the presence of iodine, iron does not seem to passivate.

7 $$\left[ {I_{2} Cl} \right]^{ - } + Fe^{0} \to Fe^{II} + 2I^{ - } + Cl^{ - }$$

8 $$\left[ {I_{3} } \right]^{ - } + Fe^{0} \to Fe^{II} + 3I^{ - }$$

**Figure 8:**
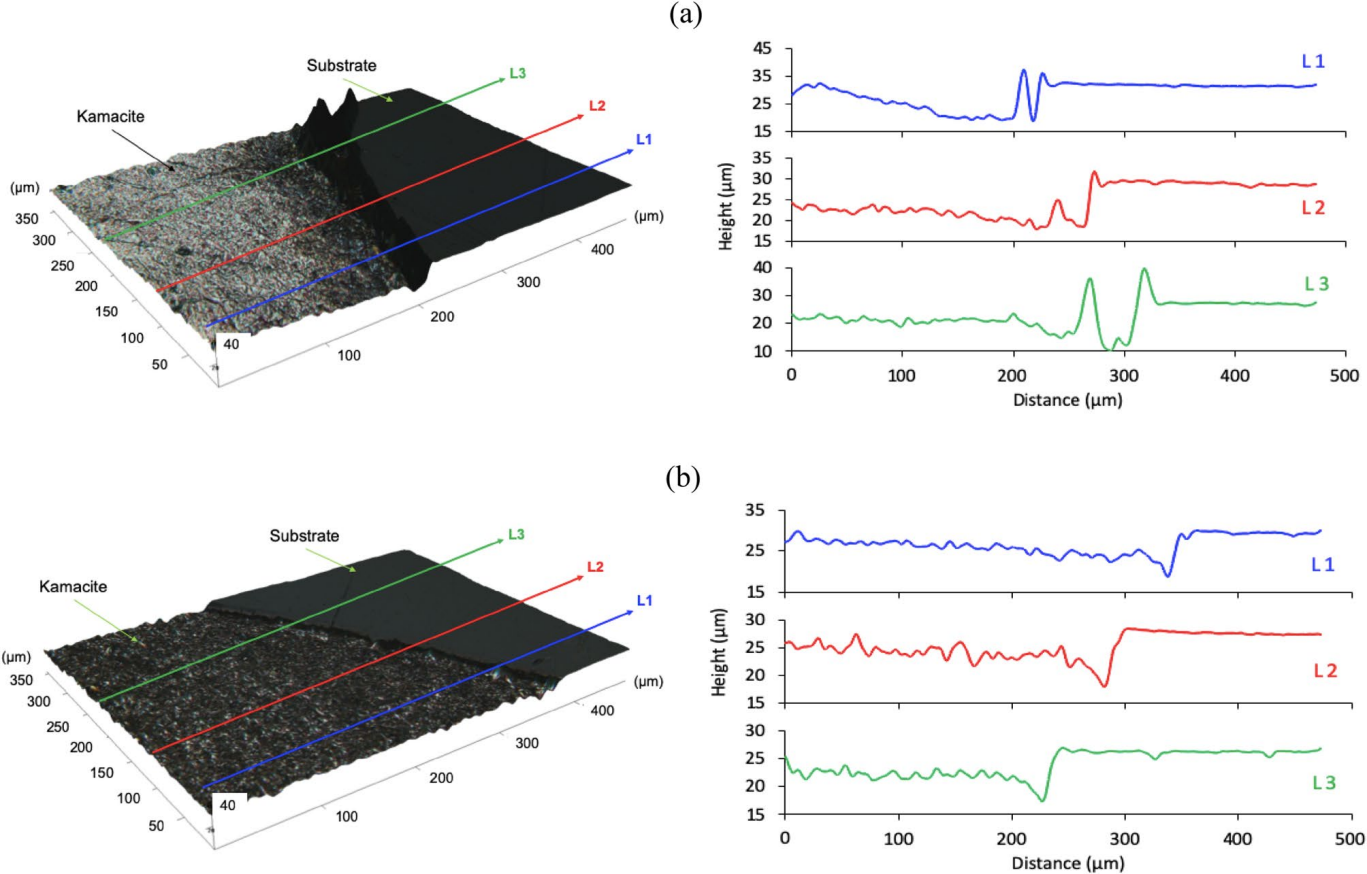
Etch depth in the Campo del Cielo sample after 60 min of etching with 0.1 mol dm^−3^ (**a**) iodine and (**b**) FeCl_3_ as oxidising agents. In the left-hand side, the 3D reflected light images (with line profiles) are described, whereas the topographic line profiles are shown in the right-hand side of the corresponding 3D image.

**Figure 9:**
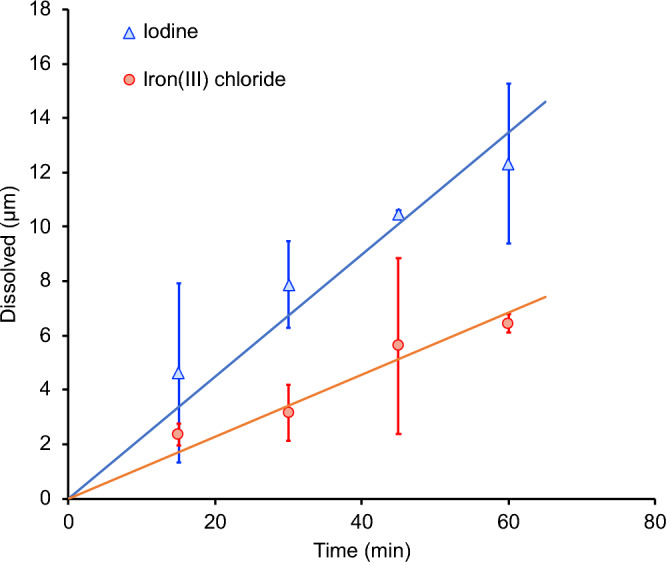
Etch depth of iron-rich phases in the Campo del Cielo meteorite with 0.1 mol dm^−3^ of iodine and FeCl_3_ as oxidising agents in the ChCl: 2EG eutectic system at 50 °C. Straight lines describe the corresponding trend. Error bars indicates the standard deviation of triplicate experiments.

The etching rate was evaluated by measuring the height profile across the sample, as shown in Fig. 8. The etch depth was measured using the level of the substrate (insoluble phase) as a reference height. As can be seen in Fig. 9, iodine allows a more efficient dissolution of metals than FeCl_3_. The average dissolution rate of the kamacite in the presence of iodine (ca. 0.22 μm min^−1^) was almost twice the dissolution rate obtained with FeCl_3_ (0.11 μm min^−1^). This confirmed that iodine is capable of providing stronger oxidising conditions than FeCl_3_. This would be expected given the difference in redox potential between these two oxidising agents. This clearly demonstrates that metal rich phases can be selectively dissolved from meteorites. In fact, the process could be further enhanced by considering electrochemical oxidation as an alternative extracting methodology. Pateli et al. have already demonstrated that the dissolution rate of iron oxide compounds increases through electrooxidation in a similar DES, while metals can be recovered on a counter electrode^36^. A similar methodology has been demonstrated for the anodic dissolution of copper sulfide minerals, and the simultaneous recovery of copper at the cathode during electrowinning^75^.

Given that metal recovery from these types of liquids has been extensively demonstrated^77,83,84^, it indicates that if a DES can be synthesised from extra-terrestrial sources or bio-produced waste, sustainable metal recovery using just solar power could be possible under extreme conditions.

## Conclusions

In this study, both iron(III) chloride and iodine were used as oxidising agents for solubilising metals from three meteorite proxies of NEAs in ChCl: 2EG deep eutectic solvent. Automated SEM–EDX analysis confirmed the chemical association of nickel with iron-rich metal phases in the original samples (before chemical etching), whose average concentration was above 10 wt.%. The mineralogical matrix in the chondrite samples was mostly composed of silicate materials, whereas the Campo del Cielo sample was constituted mostly of an iron-nickel solid solution. Some other metals of interest for space technologies such as ruthenium and rhodium were also identified as traces.

Optical profilometry was used to study the etching depth and dissolution rates in the different samples. In all samples iron-rich alloys and oxides containing nickel within their lattice structure were preferentially etched by the DES with either oxidising agent. Other metal-bearing minerals, such as troilite, did not dissolve and may have rapidly passivated. In all cases, the silicate groundmass showed no indication of etching. Etching with iodine was faster and is considered a more effective oxidising agent than iron(III) chloride for extracting target phases from meteoritic material.

## Future perspectives

Current process technologies for metal recovery and separation rely heavily on water and high-energy consumption for operation. Although the presence of water ice at both the north and south lunar poles has already been confirmed^85^, it will likely not be available for direct use as it will be used mostly as a source of oxygen and hydrogen, and so will be an expensive commodity. On the other hand, the deployment of energy-efficient methods will be crucial for the processing of materials in low-energy and ambient temperature conditions^86,87^. Thus, chemical oxidation using DESs has a great potential for metal recovery and separation from NEAs. DESs such as the eutectic system ChCl: 2EG meets some of the criteria for its potential use in space, being a largely anhydrous solvent and having a low vapour pressure. In addition, due to the relatively high chloride concentration (ca. 5 mol dm^−3^) and the absence of oxygen in space, the formation of less reactive oxide/hydroxide is circumvented.

Eventually, metals could be extracted in situ from their solid matrix(es) injecting leach solutions through injector pipes and suction from another battery of harvesting pipes, geometrically arranged to stimulate the passage of solutions through the interior of the asteroid, forcing its dissolution. Thus, pregnant leach solutions (PLS) containing the valuable metal may be retrieved at the surface. In an electrochemical cell the oxidising agent can be regenerated and simultaneously metals can be recovered from the PLS by electrowinning. It has already been demonstrated that DESs can allow the development of closed-loop flow sheets by, for instance, integrating the metallurgical unit operations of leaching and electrowinning^61,75^. Thus, dissolved metals can be selectively deposited at the cathode by adjusting the electrode potential. Although the methodology may require an artificial pressurised atmosphere to operate, energy requirements will be relatively low as the process operates at temperatures lower than 100 °C. In terms of analytical methods for mineral exploration, we have used X-ray spectra induced by electron bombardment to identify mineral phases, but other techniques may also be applicable. Laser Induced Breakdown Spectroscopy (LIBS) represents an interesting analytical tool as enables qualitative, semi-quantitative, and quantitative analysis of all elements in the periodic table. It can be performed in the laboratory or outside in the ambient environment for on-site analysis in situ, as it requires minimum sample preparation and enables a rapid online analysis^88,89^.

While in the long-term the utilisation of asteroids as mineral and metal resources is a key step in human space exploration, further investigation is required to make this a viable economic activity. The technology hereby proposed, though in a nascent stage, is very promising with DESs possessing many favourable physical and chemical characteristics and, as was demonstrated here, can selectively extract target value phases from proxy-asteroid material. Moving forward, this will require an integrated understanding of the internal structure and micro-texture of asteroids, which minerals host potentially useful metal-rich phases and their concentrations, and what electrochemical methodologies can be implemented for metal recovery.

## Material and methods

The DES was prepared from 1 mol eq of ChCl (Acros Organics, 99%) and 2 mol eq of ethylene glycol (EG, Fisher, 98%). The solvent was prepared by stirring the components at 50 °C until a colourless homogeneous liquid was formed. FeCl_3_ (Sigma Aldrich, 97%) and iodine (Fisher Scientific, > 95%) were used as oxidising agents.

Meteoritic materials used in this study were obtained from Msg-Meteorites and consist of two stony meteorites and one iron meteorite (see Fig. S1 in the Supplementary material). Stony meteorites consist of a 0.5 cm thick, approximately 6 cm square section of meteorite NWA 13876 and 5 cm long piece of meteorite NWA 7160. Both NWA meteorites belong to the high-iron (H) chemical group of ordinary chondrites. These are distinguished by their high siderophile element content, relatively small chondrules (~ 0.3 mm), and oxygen isotope compositions that are closer to the terrestrial fractionation line than those of other chondrites. The NWA 13876 is classified as type 5, as these types of meteorites have been metamorphosed at conditions that were sufficient enough to homogenise olivine and pyroxene, convert all low-calcium pyroxene to orthopyroxene, cause the growth of various secondary minerals, and blur chondrule outlines^90^. The NWA 7160 meteorite is classified as type 3, which is typically characterised by abundant chondrules, low degrees of aqueous alteration, and the presence of non-stoichiometric mineral phases^91^. The iron meteorite consists of a piece of irregularly shaped material from the Campo del Cielo meteorite. Campo del Cielo is classified as an IAB-MG type, consisting of Fe–Ni alloy that crystallised from a melt^56,92^.

All samples were documented and photographed before visually selecting potential target areas for further study. In each case an approximately 2.5 cm^2^ section was cut from the interior of the main sample, so as not to sample altered material from the fusion crust, embedded in epoxy resin and polished. Samples were imaged using a Sensofar 3D Surface Profiler to generate reflected light montages for the identification of metallic phases within the sample both prior to and post chemical etching.

Mineralogical constituents and proportions were determined by automated Scanning Electron Microscopy and Energy-dispersive X-ray spectroscopy (SEM–EDX) mineralogical analysis using a Zeiss SIGMA 200 scanning electron microscope equipped with two Bruker XFlash 6|60 energy dispersive spectroscopy detectors controlled by Zeiss Mineralogic software. Samples were imaged to provide high-resolution Back-scattered Electron (BSE) montages and analysed by EDX (accelerating voltage: 20 kV; detector aperture: 120; step size: 10 µm in NWA 13786 and NWA 7160 and 50 µm in Campo del Cielo; dwell time: 0.05 s).

In-situ etching was carried out by submerging the surface of the resin block in 80 mL DES at 50 °C with a nominal concentration of 0.1 mol dm^−3^ of oxidising agent^59^. Constant and gentle agitation was applied to maintain a homogeneous and continuous flow inside the reactor. The block was held stable with a clamp so that the stirrer bar did not contact the etching surface. The temperature was controlled using a hotplate stirrer with a glass-coated thermocouple placed directly in the etching solution.

Etching was performed step-wise for an effective period of 1 h at 50 °C. After each step, the sample was removed from the solvent, rinsed with acetone to remove any residue, and dried in air. Prior to leaching and then after each step, 2D and 3D optical images of the sample were captured by using a Zeta Instruments Zeta 2000 optical profiler using the inbuilt Zeta 3D software version 1.8.5. Images were constructed by determining the features of an image that are in focus at different heights. These were then analysed to produce a reconstructed 3D topography of the surface of each sample. After the corresponding images were obtained, the sample was returned to the solvent.

### Supplementary Information

Supplementary Information.

## Data Availability

All data generated or analysed during this study are included in this published article (and its Supplementary Information files).
