# Supplementary material for: A novel method for extracting metals from asteroids using non-aqueous deep eutectic solvents
Source: Sci Rep. 2023 Oct 8;13:16960. doi: 10.1038/s41598-023-44152-0 (PMC10560668; doi:10.1038/s41598-023-44152-0)
Supplement: Supplementary file 1 — Supplementary Information. [file 41598_2023_44152_MOESM1_ESM.docx]

**Extraterrestrial mining – A novel method for extracting minerals and metals from asteroids**

Rodolfo Marin Rivera^a*^, Philip Bird^b^, Gawen R.T. Jenkin^b^, Andrew P. Abbott^a^

^a^ *School of Chemistry, University of Leicester, Leicester, LE1 7RH*

*^b^ School of Geography, Geology and the Environment, LE1 7RH*

*^c^ School of Physics and Astronomy, LE1 7RH*

^*^ *Corresponding author. Email address: rmr26@le.ac.uk*

# Supplementary information

| (a) | (b) |
| --- | --- |
| 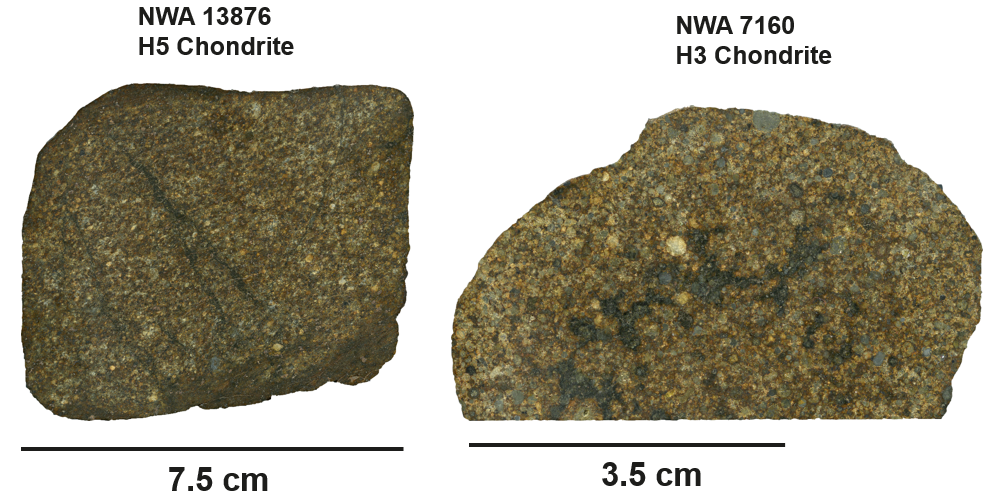 | 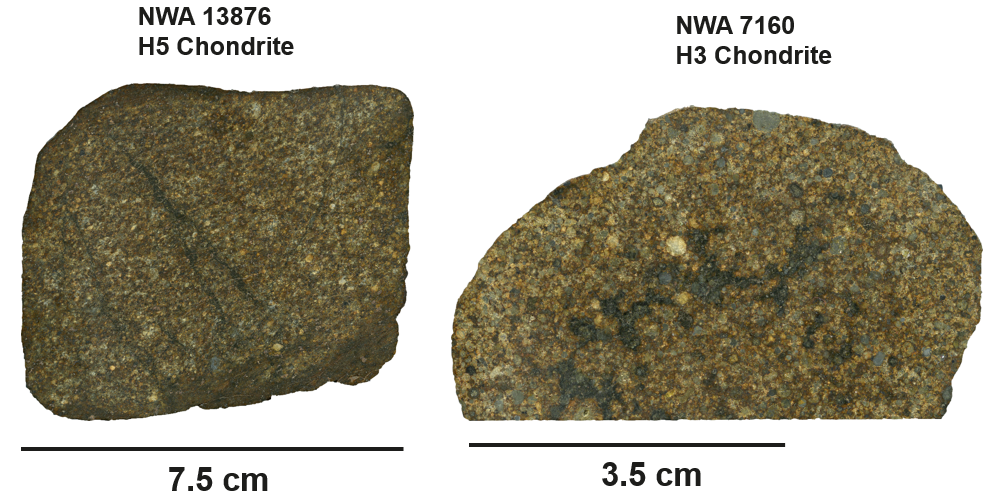 |
| (c) |  |
| 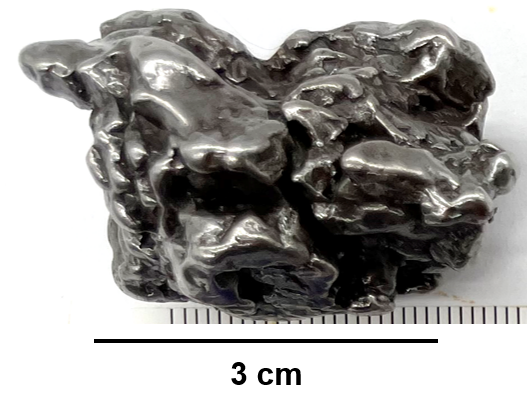 |  |

**Figure S1.** Meteorite proxies of metal-rich asteroids. (a) H5 chondrite NWA 13876, (b) H3 chondrite NWA 7160, and (c) Campo del Cielo iron meteorite.

|  |
| --- |
|  |
|  |
|  |
|  |
|  |

**Figure S2.** Element distribution in different mineral phases found in meteorites **(a)** NWA 13876, **(b)** NWA 7160 and **(c)** Campo del Cielo.

**Table S1.** Bulk analysis of meteorite NWA 13876.

| **Mineral phase** | **Area**  **(%)** | **Weight**  **(%)** | **Grain Size (µm)** | **Grain Size Std. Dev. (µm)** | **Average Composition**  **(%)** |
| --- | --- | --- | --- | --- | --- |
| Pyroxene | 37.90 | 35.96 | 60.96 | 124.22 | O 37.13; Si 29.33; Mg 19.33; Fe 13.38; Ca 0.83; |
| Augite | 0.14 | 0.12 | 18.60 | 13.80 | O 37.89; Si 28.97; Al 15.63; Na 6.54; Ca 5.97; Fe 5.01; |
| Merrillite | 0.34 | 0.26 | 36.63 | 46.71 | Ca 43.68; O 35.49; P 20.82; |
| Troilite | 4.01 | 4.62 | 42.05 | 56.93 | Fe 66.11; S 33.89; |
| (Ru-Rh) Merrillite | 0.11 | 0.09 | 42.33 | 43.00 | Ca 46.66; O 26.88; P 16.9; Ru 6.3; Rh 3.26; |
| Greigite-Violarite | 0.00 | 0.00 | 15.36 | 2.31 | Fe 54.92; S 34.85; Ni 10.23; |
| Taenite | 0.70 | 1.41 | 43.45 | 42.03 | Fe 62.77; Ni 37.23; |
| Plagioclase | 0.27 | 0.18 | 17.96 | 9.26 | O 39.08; Si 33.22; Al 15.19; Na 8.37; Ca 4.13; Fe 0; |
| Ni-Wüstite | 0.71 | 1.05 | 20.91 | 15.99 | Fe 61.29; O 31.73; Ni 6.99; |
| Wüstite | 0.38 | 0.57 | 21.44 | 17.62 | Fe 76.3; O 23.7; |
| Diopside | 9.06 | 7.29 | 33.56 | 39.74 | O 39.14; Si 29.87; Ca 12.94; Mg 10.75; Fe 7.3; |
| Kamacite | 7.25 | 14.30 | 102.59 | 138.80 | Fe 94.1; Ni 5.9; |
| Olivine | 35.82 | 34.00 | 42.55 | 86.87 | O 32.63; Mg 26.91; Fe 20.86; Si 19.59; |
| Chromite | 0.11 | 0.14 | 19.32 | 12.93 | Cr 41.36; O 24.76; Fe 24.49; Al 5.74; Mg 3.64; |

**Table S2.** Bulk analysis of meteorite NWA 7160.

| **Mineral phase** | **Area**  **(%)** | **Weight**  **(%)** | **Grain Size**  **(µm)** | **Grain Size Std. Dev.**  **(µm)** | **Average Composition**  **(%)** |
| --- | --- | --- | --- | --- | --- |
| Pyroxene | 27.18 | 27.64 | 43.84 | 81.39 | O 36.83; Si 29.11; Mg 18.72; Fe 14.32; Ca 1.02; |
| Taenite | 0.13 | 0.29 | 17.69 | 8.14 | Fe 73.5; Ni 26.5; |
| Kamacite | 1.96 | 4.15 | 57.98 | 72.55 | Fe 88.28; Ni 11.72; |
| Wüstite | 0.54 | 0.87 | 21.64 | 18.74 | Fe 74.3; O 25.7; |
| Merrillite | 0.11 | 0.09 | 21.41 | 15.23 | Ca 43.34; O 36; P 20.66; |
| (Ru-Rh) Merrillite | 0.02 | 0.02 | 31.46 | 22.55 | Ca 47.18; O 28.03; P 17.39; Ru 4.88; Rh 2.51; |
| Greigite-Violarite | 0.04 | 0.05 | 19.12 | 10.11 | Fe 53.62; S 36.11; Ni 10.27; |
| Augite | 0.06 | 0.06 | 16.28 | 5.27 | O 37.96; Si 26.94; Al 16.48; Ca 8.08; Na 5.74; Fe 4.79; |
| Ni-Wüstite | 4.17 | 6.67 | 27.85 | 25.78 | Fe 56.87; O 35.73; Ni 7.39; |
| Diopside | 10.95 | 9.44 | 33.98 | 35.54 | O 38.9; Si 28.93; Ca 11.34; Fe 10.46; Mg 10.37; |
| Troilite | 3.73 | 4.61 | 40.48 | 59.37 | Fe 65.45; S 34.55; |
| Plagioclase | 0.19 | 0.13 | 20.04 | 14.21 | O 38.28; Si 29.1; Al 17.71; Ca 8.7; Na 6.2; Fe 0; |
| Olivine | 45.19 | 45.96 | 37.14 | 75.34 | O 31.96; Fe 26.52; Mg 22.9; Si 18.62; |
| Chromite | 0.02 | 0.03 | 16.95 | 6.60 | Cr 37.48; O 25.86; Fe 24.05; Al 8.44; Mg 4.16; |
| Unclassified | 5.71 | 0.00 | 29.94 | 23.16 | O 28.29; Fe 24.92; Si 20.17; Mg 6.15; Al 5.31; Ca 4.1; S 4.08; Na 2.99; Ni 1.55; Cr 1.35; P 0.52; K 0.25; Ru 0.1; Ti 0.06; Re 0.04; Rh 0.04; Mn 0.02; Ag 0.01; Cu 0.01; Sn 0.01; Bi 0.01; Se 0.01; |

**Table S3.** Element distribution in meteorite Campo del Cielo.

| **Mineral phase** | **Area**  **(%)** | **Weight**  **(%)** | **Grain Size**  **(µm)** | **Grain Size Std. Dev.**  **(µm)** | **Average Composition**  **(%)** |
| --- | --- | --- | --- | --- | --- |
| Kamacite | 98.56 | 98.99 | 1420.37 | 698.47 | Fe 94.91; Ni 5.09; |
| Wüstite | 0.37 | 0.28 | 76.36 | 57.75 | Fe 77.95; O 22.05; |
| Ni-Wüstite | 0.05 | 0.04 | 59.36 | 22.17 | Fe 66.8; O 26.6; Ni 6.6; |
| Taenite | 0.66 | 0.68 | 72.50 | 92.19 | Fe 65.61; Ni 34.39; |
| Unclassified | 0.36 | 0 | 79.59 | 55.30 | Fe 44.11; O 43.52; Si 4.75; Ni 3.26; Al 0.94; P 0.65; Na 0.43; Ru 0.39; Bi 0.3; Mg 0.22; Ge 0.17; Ca 0.16; Ir 0.15; S 0.15; Pt 0.12; Se 0.1; Zn 0.07; Pb 0.06; Pd 0.06; Ba 0.06; Au 0.05; Cr 0.05; Ag 0.04; Rh 0.04; Zr 0.04; Ti 0.03; Hg 0.03; Rb 0.02; Sn 0.01; |


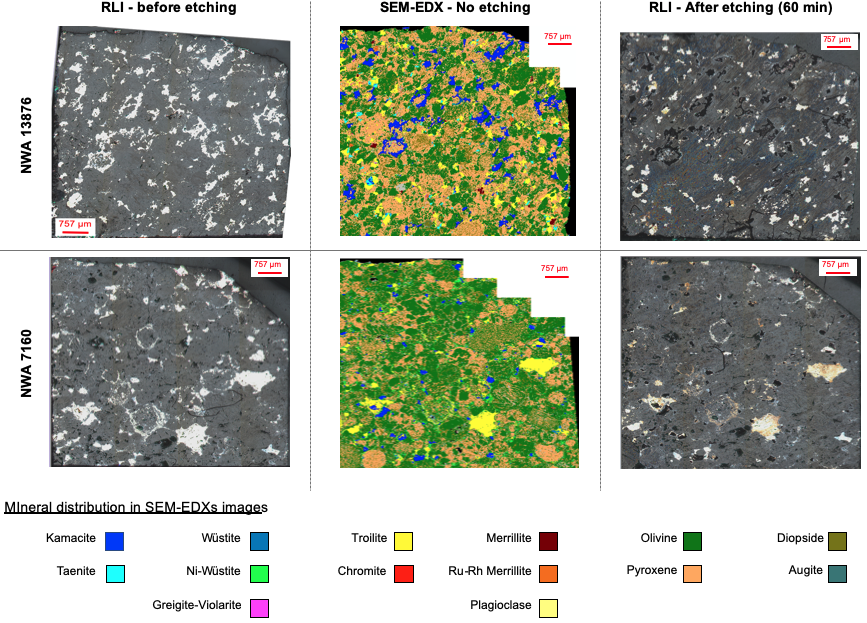


**Figure S3.** Reflected light images of meteorites NWA 13876 and NWA 7160 before and after 60 min of chemical etching with 0.1 mol dm ^̶ 3^ of iodine as oxidising agent in ChCl: 2EG eutectic solvent. The distribution of minerals is depicted in the corresponding SEM-EDX images.


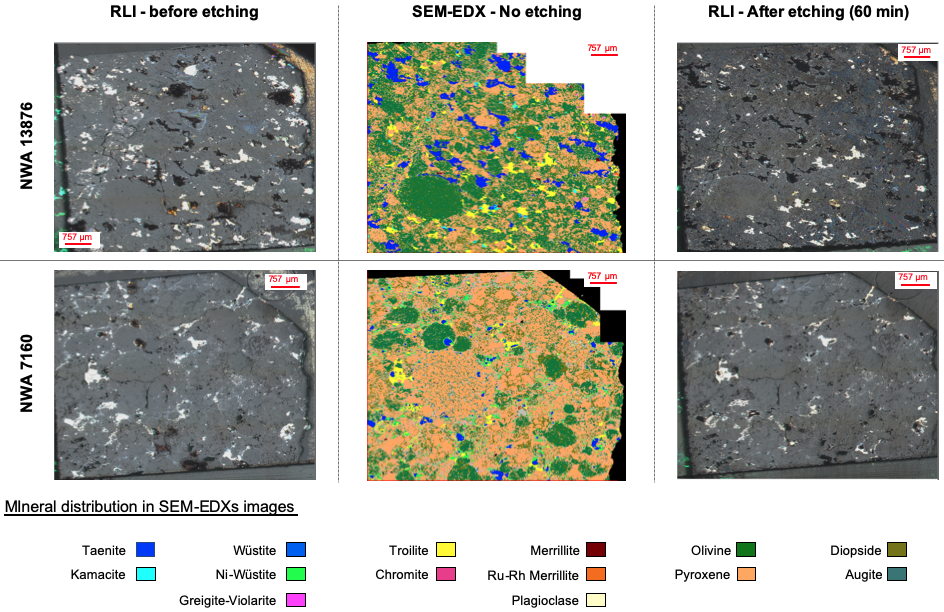


**Figure S4.** Reflected light images of meteorites NWA 13876 and NWA 7160 before and after 60 min of chemical etching with 0.1 mol dm ^̶ 3^ of FeCl_3_ as oxidising agent in ChCl: 2EG eutectic solvent. The distribution of minerals is depicted in the corresponding SEM-EDX images.
